# Prevalencia de malaria gestacional en Ecuador

**DOI:** 10.7705/biomedica.6184

**Published:** 2022-03-01

**Authors:** Ángela Bracho, María Leonela Guerrero, Gema Molina, Zulbey Rivero, Miguel Arteaga

**Affiliations:** 1 Cátedra de Parasitología, Universidad Técnica de Manabí, Portoviejo, Ecuador Universidad Técnica de Manabí Universidad Técnica de Manabí Portoviejo Ecuador; 2 Licenciatura en Laboratorio Clínico, Universidad Técnica de Manabí, Portoviejo, Ecuador Universidad Técnica de Manabí Licenciatura en Laboratorio Clínico Universidad Técnica de Manabí Portoviejo Ecuador; 3 Práctica Profesional de Parasitología, Universidad del Zulia, Maracaibo, Venezuela Universidad del Zulia Universidad del Zulia Maracaibo Venezuela

**Keywords:** Plasmodium vivax, Plasmodium falciparum, malaria, transmisión vertical de enfermedad infecciosa, Ecuador, Plasmodium vivax, Plasmodium falciparum, malaria, infectious disease transmission, vertical, Ecuador

## Abstract

**Introducción.:**

La malaria gestacional, definida como la presencia de *Plasmodium* spp. en sangre periférica materna o el hallazgo del parásito en la placenta, es considerada un importante problema de salud pública en las regiones tropicales y subtropicales.

**Objetivo.:**

Determinar la frecuencia de casos de malaria gestacional diagnosticados en Ecuador entre 2015 y 2018.

**Materiales y métodos.:**

Se hizo un estudio descriptivo, retrospectivo y transversal.

**Resultados.:**

Se determinaron 46 casos de malaria gestacional en el período evaluado, 25 por *Plasmodium falciparum* y 21 por *Plasmodium vivax*, siendo el 2018 el año con más casos. En cuanto a las variables de edad y trimestre de gestación, prevalecieron en el grupo de 20 a 29 años (46 %) y en el segundo trimestre (37 %). Solo se observó una diferencia significativa entre los casos por año y la especie parasitaria.

**Conclusión.:**

La malaria gestacional en Ecuador ha aumentado en los últimos cinco años, por lo que es importante informar a las mujeres en estado de gravidez sobre las medidas preventivas para evitar el contagio con el parásito, dadas las graves consecuencias que conlleva para ellas y sus hijos.

## Abstract (en)

**Introduction:**

Gestational malaria, defined as the presence of *Plasmodium* spp. in maternal peripheral blood or in the placenta, is considered an important public health problem in tropical and subtropical regions.

**Objective:**

To determine the frequency of gestational malaria cases diagnosed in Ecuador between 2015 and 2018.

**Materials and methods:**

We conducted a descriptive, retrospective, and cross-sectional study.

**Results:**

There were 46 cases of gestational malaria between 2015 and 2018: 25 caused by *Plasmodium falciparum* and 21 by *Plasmodium vivax*. The year with the most cases in this period was 2018. The age group most affected was 20 to 29 years old with 21 cases (46%). Prevalence was found to be highest in the second trimester of pregnancy with 17 cases (37%). A significant difference was only observed between cases per year and parasitic species.

**Conclusion:**

The prevalence of gestational malaria in Ecuador increased in the last five years. Therefore, it is important to inform pregnant women about preventive measures to avoid infection given its serious consequences both for the mother and her unborn child.

La malaria o paludismo gestacional se ha definido como la presencia de *Plasmodium* spp. en sangre periférica materna o el hallazgo del parásito en la placenta ^1^. La infección por malaria durante el embarazo es un importante problema de salud pública en las regiones tropicales y subtropicales del mundo y, dada la inmunodepresión secundaria por el embarazo, las mujeres gestantes son más vulnerables ante la enfermedad, con consecuencias tanto para ellas como para sus hijos ^2^.

Durante el embarazo, el paludismo puede provocar el nacimiento de niños con bajo peso y producir retraso en el crecimiento intrauterino del feto, entre otros trastornos. Las mujeres embarazadas con una inmunidad adquirida contra la malaria relativamente baja, están en mayor riesgo de sufrir las peores complicaciones, entre ellas, anemia grave por malaria, aborto, nacimiento de un niño muerto, o muerte de la madre y del recién nacido. El grado de endemicidad de la transmisión de la malaria, el grado de inmunidad adquirida y el número de embarazos anteriores, son algunos de los principales factores que influyen en la epidemiología de la malaria durante el embarazo ^3^.

La malaria en Ecuador ha sido uno de los mayores problemas de salud pública en vastas extensiones del territorio, con focos endémicos de alta transmisión que han persistido especialmente en el norte del litoral y el norte de la Amazonía, y favorecen su dispersión hacia zonas vecinas con condiciones de cierto deterioro epidemiológico que, además, enfrentan cada vez con mayor frecuencia fenómenos climáticos adversos ^4^.

En el 2008, Ecuador fue el país con menor incidencia de malaria y menor número de casos por *P. falciparum* entre los países endémicos de la región amazónica. En ese período, se registraron 4.986 casos de la enfermedad, es decir, una incidencia de 0,4 casos por 1.000 habitantes, un índice de paludismo (IPA) próximo al registrado en varios de los países de Centroamérica ^5^.

En el informe de la Organización Mundial de la Salud (OMS) sobre el paludismo en el 2019, se estimaban 229 millones de casos a nivel mundial y 409.000 fallecimientos ^6^. En Ecuador, se han hecho importantes esfuerzos para el control de la malaria, lo que se ha visto reflejado en el descenso del número de casos desde el 2002. Sin embargo, según el informe de casos reportados por provincia entre el 2014 y el 2017 en el Sistema de Vigilancia Epidemiológica de Malaria del Ecuador (SIVEMAE), ha habido un incremento de casos ocasionados por las dos especies circulantes, *P. vivax* y *P. falciparum*, a pesar de lo cual, el país ha logrado convertirse en uno de los 21 países que ya marchan hacia la eliminación del paludismo ^7^.

En este contexto, se decidió hacer el presente estudio para determinar la frecuencia de casos de malaria gestacional diagnosticados en Ecuador entre el 2015 y el 2018.

## Materiales y métodos

Se hizo un estudio descriptivo, retrospectivo y transversal.

### Población y muestra

La población de estudio incluyó a 293.139 mujeres embarazadas en el período de 2015 a 2018 en Ecuador según los datos del Instituto Nacional de Estadística y Censos (INEC). Teniendo en cuenta el número total de las provincias con casos positivos de malaria gestacional en el país, el tamaño de la muestra correspondió a 46 mujeres embarazadas.

### Análisis de los datos

La recolección de los datos contó con la autorización de la coordinación de la zonal 4 de vigilancia en salud adscrita al Ministerio de Salud Pública, lo que permitió determinar las zonas en la que se presentaron casos diagnosticados como positivos de malaria gestacional en el país. El diagnóstico del laboratorio de paludismo a nivel nacional se hace inicialmente mediante pruebas de diagnóstico rápido (PDR) y, posteriormente, los casos positivos son confirmados mediante gota gruesa y extendido coloreado por personal del Ministerio de Salud Pública.

### Análisis estadístico

Se utilizó el programa estadístico SPSS, versión 17 (SPSS Inc., Chicago, III, USA) y la prueba de ji al cuadrado con un nivel de significación del 95 % y un valor de p de 0,05 o menos para determinar las diferencias significativas.

### Aspectos éticos

El estudio cumplió con las normas y principios éticos, establecidos y aprobados por el Comité de Bioética de la Facultad de Ciencias de la Salud de la Universidad Técnica de Manabí, incluida la declaración sobre ausencia de conflictos de intereses y el acuerdo de confidencialidad sobre los datos y resultados (número de aprobación: PTL-21-19).

## Resultados

En el periodo estudiado, los casos de malaria gestacional diagnosticados se distribuyeron así: en el 2015, 4 casos (8,7 %), en el 2016, 7 casos (15,2 %), en el 2017, 14 casos (30,4 %) y en el 2018, 21 casos (45,7 %), evidenciándose así un aumento exponencial.

La edad de las pacientes estuvo comprendida entre los 15 y los 49 años (media=25,4; desviación estándar, DE=7,2; mediana=24,5), con una mayor frecuencia en el grupo etario de 20 a 29 años (46 %), seguido por el de mujeres gestantes de 15 a 19 años (28 %), y en último lugar, mujeres entre los 40 y 49 años (4 %). En cuanto al grupo étnico, 25 eran indígenas (54,35 %), 13 mestizas (28,26 %) y 8 afrodescendientes (17,39 %).

En cuanto a la ocupación, el 37 % se dedicaba a los oficios del hogar y el 34,8 % correspondía a trabajadoras domésticas, en tanto que las estudiantes representaron el 15 %, las agricultoras, 9 %, y, con un porcentaje más bajo, las estilistas, con el 2%.

Según los datos relativos a la especie causante de la infección, la mayor prevalencia fue la de *P. falciparum,* con el 54,35 % (25 casos), en tanto que el 45,65 % (21 casos) correspondió a *P. vivax.*

No se encontraron diferencias significativas entre las variables de grupo etario y especie, ni entre el trimestre de gestación y la especie (p>0,05). La incidencia en el segundo trimestre fue del 37 %. Los casos de *P. vivax* fueron más predominantes en el segundo y tercer trimestres, y los de *P. falciparum* fueron más frecuentes en el primero y segundo trimestres. Como se observa en el cuadro 1, se encontró una diferencia significativa al correlacionar el año de estudio con la presencia de *P. vivax* o *P. falciparum*.

**Cuadro 1:** Características estudiadas y su relación con la frecuencia por especie encontrada

Características			P. vivax* (n=21)		***P. falciparum* (n=25)**		Total (n=46)	
			n	%	n	%	n	%
Periodo de registro de casos*								
	2018		21	100	0	0	21	45,7
	2017		0	0	14	56,0	14	30,4
	2016		0	0	7	28,0	7	15,2
	2015		0	0	4	16,0	4	8,7
Grupos de edad (años)								
	15-19		6	28,6	7	28,0	13	28,3
	20-29		9	42,9	12	48,0	21	45,7
	30-39		4	19,04	6	24,0	10	21,7
	40-49		2	2	0	0	2	4,3
Edad gestacional (trimestre)								
	1-13 (primero)		5	23,8	9	36,0	14	30,4
	14-25 (segundo		8	38,1	9	36,0	17	37,0
	26-39 (tercero)		8	38,1	7	28,0	15	32,6
Ocupación								
	Ama de casa		6	28,6	11	44	17	37
	Empleada doméstica		7	33,3	9	36	16	34,8
	Estudiante		5	23,8	2	8	7	15,2
	Agricultor		2	9,5	2	8	4	8,7
	Conchera		0	0	1	4	1	2,2
	Estilista		1	4,8	0	0	2	2,2

* p<0,001; grados de libertad = 3 (diferencia significativa)

Se evidenció una mayor frecuencia de *P. falciparum* entre las amas de casa y las empleadas domésticas comparadas con el resto de las ocupaciones. Entre las estudiantes, se observó una mayor prevalencia de *P. vivax* (23,8 %) que de *P. falciparum* (8,0 %). No obstante, los análisis de asociación mediante la prueba de ji al cuadrado de Pearson, no evidenciaron diferencias significativas (p=0,454; grados de libertad, gl=5) entre la ocupación y la especie parasitaria.

**Figura 1:**
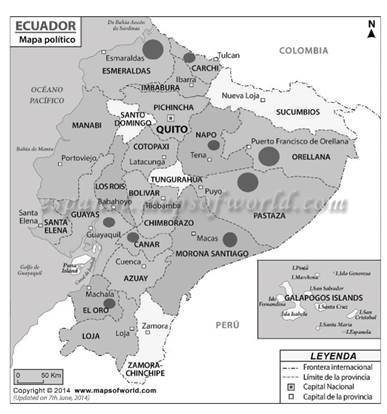
Mapa de Ecuador con la distribución de casos con malaria gestacional, 2015-2018

Se determinó que la mayor frecuencia de casos de paludismo gestacional en el Ecuador se produjo en la provincia de Esmeraldas, con 16 casos (35 %), seguido de Pastaza con 11 casos (24 %) y Orellana con 10 casos (22 %), en tanto que las demás provincias obtuvieron un bajo porcentaje de casos, con 4 casos (9 %) en Morona Santiago y 1 caso (2 %) en cada uno las provincias de Carchi, Napo, Cañar, El Oro y Guayas (figura 1).

## Discusión

Se evidenció un aumento en los casos de malaria gestacional en el país durante el periodo de estudio, lo que coincide con el estudio de Jadan, *et al*. ^5^, quienes señalan que, desde el 2017 hasta la actualidad, ha habido una tendencia al incremento de la enfermedad en algunas regiones endémicas, lo que constituye no solo un problema de salud, sino de desarrollo social y económico.

Purizaca ^8^ señala que estas cifras de prevalencia se han mantenido en el tiempo y que existe un riesgo de incremento en los próximos años debido al aumento de la resistencia a las drogas antipalúdicas y el cambio climático. En contraste, Nogueira, *et al.*^9^, encontraron en su estudio en Porto Velho, Brasil, en el cual compararon el número de casos de malaria gestacional entre el 2014 y el 2018, una tendencia a la disminución en la distribución anual.

En cuanto a la edad, es importante señalar que es un factor de riesgo de malaria gestacional, hecho ya constatado por otros investigadores ^5^^,^^10^^,^^11^, quienes señalan que, aunque la malaria afecta a personas de todas las edades, la mayoría de los casos diagnosticados en la región se registran en personas entre los 15 y los 49 años de edad, es decir, las etapas de mayor productividad económica de la vida, datos que coinciden con el porcentaje obtenido en el presente estudio. Asimismo, en varios estudios ^12^^-^^17^ en mujeres embarazadas, se ha evidenciado el predominio del grupo de 15 a 29 años, probablemente por el hecho de que, a esa edad, las mujeres están sexualmente activas y la posibilidad del embarazo es mayor.

Desde el punto de vista de la vigilancia epidemiológica para el diagnóstico de la malaria, es importante clasificar el caso como autóctono o importado. La importancia radica en que los casos importados de malaria de países vecinos también pueden jugar un papel importante en la transmisión de la malaria en zonas que no son consideradas endémicas, o donde la prevalencia es baja, situación demostrada en Brasil por Carlos, *et al.*^18^, cuando comprobaron que los casos en el estado de Maranhão y Roraima provenían principalmente de Venezuela y Guayana, siendo los municipios más afectados aquellos fronterizos con estos países.

En el mismo contexto, la movilización a zonas endémicas dentro del país o entre países vecinos indudablemente influye en esa situación. Entre los datos obtenidos, se menciona que hubo movilización de las mujeres por las provincias de Pastaza, Minas de San José de Cachavi, Quininde y Colombia, en tanto que, en el caso de Venezuela, provenían de Ciudad Bolívar (estado Bolívar), la cual es una zona de riesgo en ese país ^19^. Así pues, la nacionalidad de los pacientes afectados puede influir, pero, a pesar de la importante migración que existe actualmente en el país, se reportaron 37 casos autóctonos y 9 importados, cantidades que correspondieron a la nacionalidad ecuatoriana y otras, respectivamente. Esta situación podría explicarse ya que las mujeres extranjeras que ingresan a Ecuador no cuentan con estabilidad económica o laboral en este país, por lo que evitan quedar embarazadas.

En este sentido, Jadan, *et al.*^5^, han señalado que el aumento pronunciado de casos importados en los últimos años, es otro riesgo que debe tenerse en cuenta y subraya la necesidad de una mayor cooperación con los países vecinos. En Ecuador, las zonas maláricas se concentran principalmente en unas pocas provincias a lo largo de la frontera amazónica con Perú y en la zona costera noroccidental fronteriza con Colombia, lo cual coincide con los resultados presentados aquí, ya que 8 de los casos procedían de ese país. Por otra parte, Murillo, *et al*. ^20^, precisan que durante los últimos seis años se reportaron en Colombia 294 casos importados de malaria, lo que demuestra que la migración sigue siendo un factor importante en la aparición de estas enfermedades.

No hay muchos artículos en los que se relacione la ocupación con el paludismo en mujeres embarazadas. Sin embargo, López-Pérez, *et al.*^17^, informaron en su estudio que 30 de 34 mujeres trabajaban en casas; por su parte, Feleke, *et al.*^16^, encontraron entre las ocupaciones la de agricultora, comerciante, empleada de gobierno y ama de casa, siendo esta última la de mayor frecuencia (39,2 %). Algunos estudios en diferentes poblaciones demuestran que la malaria es una enfermedad asociada a las condiciones de trabajo. En general, es más común en las áreas rurales y en labores relacionadas con la agricultura como, por ejemplo, el cultivo del arroz ^21^.

Balami, *et al.*, entre los factores de riesgo de la malaria en mujeres embarazadas, refieren factores sociodemográficos como la edad, el nivel educativo, los ingresos y el empleo, señalando que las mujeres con un mayor nivel de educación tienen menos probabilidades de adquirir malaria, lo que indicaría una asociación entre la ocupación y esta enfermedad, dado que la mayoría de aquellas que se dedican a labores domésticas y oficios del hogar tienen un bajo nivel educativo ^22^.

En el presente estudio, las amas de casa y las trabajadoras domésticas fueron las más afectadas por la malaria y es posible inferir que, al trabajar en casas, tal vez no toman las precauciones necesarias por desconocimiento, lo que aumenta las posibilidades de contacto con el vector y, por ende, de contraer la enfermedad.

Según nuestros datos, los casos más frecuentes correspondieron a los causados por *P. falciparum,* con 54,35 %, en tanto que el 45,65 % correspondió a *P. vivax*, lo que concuerda con los resultados de otros estudios en Ecuador, como el de Vera-Arias, *et al*. ^23^*.* Asimismo, los resultados sugieren que, en las regiones amazónica y costera, específicamente en la costa noroeste del Ecuador, zona que, al igual que en Colombia ^17^, ha sido históricamente endémica para *P. falciparum*, también se ha registrado un aumento de casos por esta especie. En contraste, en algunos estudios en otros países como Venezuela ^24^, Honduras ^12^ y Brasil ^9^, predominaron los casos causados por *P. vivax* e, incluso, hubo infecciones mixtas. *Plasmodium falciparum* se ha asociado mayormente con los casos de paludismo gestacional debido a su gran potencial de infección, como lo reportan diversos estudios ^9^^,^^15^^,^^25^, aunque las mujeres embarazadas no se escapan de adquirir la infección por *P. vivax*, como lo reportaron recientemente Brummaier, *et al.*^26^, quienes describen las múltiples condiciones específicas que sufre la mujer infectada por esta especie.

La información sobre la estructura genética de la población de *P. falciparum* en Ecuador es limitada ^23^. Un estudio molecular de *P. falciparum* durante un brote en la ciudad de Esmeraldas entre noviembre de 2012 y noviembre de 2013, reveló que los parásitos fueron el resultado de una expansión clonal de *P. falciparum* que circulaba en niveles bajos o había invadido a Ecuador, procedente de países fronterizos ^23^^,^^27^.

Algunos estudios señalan que la frecuencia de la malaria gestacional varía según la edad gestacional ^28^. Nuestros resultados coinciden con los de otros estudios ^9^^,^^16^^,^^17^^,^^24^ en los que se evidenció una mayor incidencia en el segundo trimestre, aunque sin diferencias significativas, pero difieren de lo obtenido por Carmona, *et al.*^14^, quienes encontraron que los casos aumentaban a medida que se incrementaba la edad gestacional.

En los estudios de Fernández, *et al.*^12^, Jarude, *et al*. ^28^, y Santos, *et al*. ^29^, se reportó un aumento de casos en el tercer trimestre del embarazo, en tanto que Adam, *et al.*^13^, encontraron que la edad gestacional no tenía relación con la adquisición de la enfermedad.

Nuestros resultados sugieren que la mujer embarazada puede ser más propensa a contraer la enfermedad en el segundo trimestre del embarazo, cuando parece darse la tasa más alta de infección, lo que enfatiza la necesidad de brindar atención previa al parto como parte de los esfuerzos de prevención, control y tratamiento de la malaria ^9^.

A pesar de que no se registró aquí la sintomatología de las mujeres estudiadas, es importante mencionar que la edad gestacional influye en la aparición de los síntomas clínicos y sus complicaciones. Algunos estudios han relacionado la anemia con la infección malárica, independientemente de la especie, en tanto que la malaria gestacional producida por *P. falciparum* se ha asociado con manifestaciones clínicas y complicaciones que aumentan el riesgo de muerte en la embarazada ^2^. Por su parte, Piñeros, *et al.*^1^, indican que la mayor parasitemia durante el segundo trimestre de gestación se correlacionó con una mayor presencia de síntomas clínicos.

En cuanto a las zonas de riesgo para el paludismo en Ecuador, Pastaza, Orellana, Morona Santiago, Cotopaxi y Esmeraldas ^30^, los resultados aquí reportados reiteran que estas provincias son las más afectadas por malaria gestacional y, aunque se encontró un bajo porcentaje en la provincia El Oro, es importante mencionar que, con Esmeraldas, constituye un paso fronterizo, lo que influye en la trasmisión de la enfermedad a partir del paso de personas entre los países vecinos.

A pesar de los logros en la batalla contra esta enfermedad, Ecuador todavía tiene focos de transmisión activos, donde la incidencia de malaria no ha disminuido en comparación con otras regiones del país. Tal es el caso del cantón San Lorenzo (Esmeraldas), cuya población, servicios de salud y factores ecoepidemiológicos presentan condiciones particulares que hacen que la reducción de los casos de malaria sea incierta ^31^^,^^32^, siendo este el cantón más afectado por casos de malaria gestacional.

En conclusión, se registraron 46 casos de malaria gestacional en Ecuador entre el 2015 y el 2018, siendo este último el año el de mayor número de casos. Es recomendable que, en las zonas de riesgo del país, se haga más énfasis en la promoción de salud, de manera que las mujeres, sobre todo las que desempeñan labores en el hogar, mantengan las medidas preventivas adecuadas para evitar el contagio, ya que la infección puede traer graves consecuencias para ellas y sus hijos.
